# Supplementary material for: Rice growth and yield responses to saline water irrigation are related to Na+/K+ ratio in plants
Source: PLoS One. 2024 Nov 1;19(11):e0312372. doi: 10.1371/journal.pone.0312372 (PMC11530064; doi:10.1371/journal.pone.0312372)
Supplement: S1 Data — (DOCX) [file pone.0312372.s001.docx]

Rice growth and yield responses to saline water irrigation are related to Na^+^/K^+^ ratio in plants

Priya Lal Chandra Paul^1*^, Afsana Jahan^2^, Palash Kumar kundu^1^, Debjit Roy^1^, Richard W Bell^3^, Belal Hossain^1^, Rakiba Sultana^4^, Mohammad Rezoan Bin Hafiz Pranto^1^, Tanjina Islam^2^, Sharon E Benes^5^, Md Rafiqul Islam^2^

*^1^ Irrigation and Water Management Division, Bangladesh Rice Research Institute, Gazipur-1701, Bangladesh*

^2^ Soil Science Division, *Bangladesh Rice Research Institute, Gazipur-1701, Bangladesh*

^3^Centre for Sustainable Farming Systems, Future Food Institute, Murdoch University, WA-6150, Australia

*^4^Agronomy Division, Bangladesh Rice Research Institute, Gazipur-1701, Bangladesh*

^5^Department of Plant Science, California State University, Fresno, CA 93740-8033

*^*^Corresponding author: Tel.: +8801716617378; E-mail:* [*plcpauliwm@yahoo.com*](mailto:plcpauliwm@yahoo.com)

**Supplementary materials**

|  | **Mean value of different variable under the treatments of saline wate irrigation and varieties** | | | | | | | | | | | | | | | | | |
| --- | --- | --- | --- | --- | --- | --- | --- | --- | --- | --- | --- | --- | --- | --- | --- | --- | --- | --- |
| Variety | Saline water irrigation | Grain weight (g) | Filled grain (no) | Unfilled grain (no) | Fertility (%) | Tiller number (no) | Straw weight (g) | Root weight (g) | EC1:5 at 45 days | SP at 45 days | EC1:5 at harvest | SP at harvest | K (mmol kg-1) in straw | Na (mmol kg-1) in straw | Cl (mmol kg-1) in straw | K (mmol kg-1) in root | Na (mmol kg-1) in root | Cl (mmol kg-1) in root |
| V1 | I1 | 34.73 | 1654 | 8.47 | 96.54 | 28.33 | 27.21 | 3.31 | 0.16 | 160.4 | 0.21 | 207.4 | 454.82 | 387.94 | 757.70 | 656.46 | 204.76 | 1356.42 |
| V1 | I2 | 29.40 | 1400 | 23.15 | 95.39 | 26.67 | 23.55 | 2.27 | 0.29 | 279.1 | 0.43 | 451.3 | 416.18 | 475.32 | 1353.01 | 648.01 | 280.30 | 2213.34 |
| V1 | I3 | 18.48 | 880 | 26.65 | 91.15 | 16.33 | 11.73 | 1.32 | 0.39 | 383.3 | 0.49 | 510.7 | 432.16 | 763.39 | 1461.69 | 599.12 | 310.52 | 2414.15 |
| V1 | I4 | 7.56 | 360 | 31.23 | 70.68 | 7.67 | 6.24 | 0.92 | 0.41 | 427.8 | 0.89 | 924.2 | 349.76 | 1041.39 | 1765.02 | 541.63 | 282.61 | 2435.12 |
| V1 | I5 | 4.06 | 193.33 | 57.67 | 49.36 | 5.00 | 3.67 | 0.85 | 0.41 | 448.8 | 1.40 | 1476 | 336.30 | 1398.67 | 1624.93 | 486.83 | 345.94 | 2653.94 |
| V2 | I1 | 35.21 | 1676.6 | 7.93 | 99.53 | 30.00 | 31.09 | 4.73 | 0.15 | 153.4 | 0.16 | 186 | 404.40 | 365.11 | 619.01 | 623.00 | 269.27 | 1446.59 |
| V2 | I2 | 30.03 | 1430 | 22.53 | 98.45 | 27.67 | 27.77 | 3.47 | 0.26 | 269.2 | 0.39 | 411 | 434.70 | 578.90 | 1415.40 | 552.45 | 328.39 | 1995.36 |
| V2 | I3 | 19.60 | 933.33 | 26.30 | 95.91 | 17.33 | 20.66 | 2.07 | 0.37 | 368.2 | 0.49 | 510 | 406.77 | 781.83 | 1612.25 | 573.25 | 312.15 | 2520.88 |
| V2 | I4 | 8.51 | 405.33 | 32.37 | 89.83 | 8.33 | 11.10 | 1.87 | 0.40 | 415.8 | 0.91 | 968 | 345.75 | 775.97 | 1384.72 | 562.42 | 350.48 | 2213.58 |
| V2 | I5 | 5.18 | 246.67 | 60.70 | 80.24 | 6.67 | 6.97 | 0.96 | 0.41 | 420.9 | 1.20 | 1365 | 333.08 | 976.40 | 1598.12 | 549.83 | 451.32 | 2735.67 |
