# Supplementary material for: Rice growth and yield responses to saline water irrigation are related to Na+/K+ ratio in plants
Source: PLoS One. 2024 Nov 1;19(11):e0312372. doi: 10.1371/journal.pone.0312372 (PMC11530064; doi:10.1371/journal.pone.0312372)
Supplement: Attachment — Submitted filename: review.docx [file pone.0312372.s002.docx]

This manuscript is well-structured, the methods are sound, the results are comprehensive, and the discussion provides a thoughtful interpretation of the findings. The study contributes to the understanding of the physiological and ionic responses of rice to long-term saline water irrigation, which is relevant for addressing the challenges of salinity in rice production. However, I suggest minor revisions that would help to improve the clarity, flow, and overall presentation of the manuscript.

**Title:**

The current title "Rice growth and yield responses to saline water irrigation are caused by increased ion concentration in straw and roots" is a bit lengthy and technical. Consider a more concise and clear title, such as:

**Introduction:**

- Consider adding a sentence or two to provide more context on the use of saline water for rice cultivation in specific regions, such as the Ganges Delta, where this study was conducted.

- You could also briefly mention the importance of understanding the physiological and ionic mechanisms behind the response of different rice cultivars to long-term saline water irrigation.

**Materials and Methods:**

- in the sentence “……….were analyzed using 2-way ANOVAs” ANOVAs should be ANOVA, as it is one analysis or one design not two ANOVA designs.

- “………were separated by least significant difference (LSD) at *P* = 0.05 ” ) … *P* = 0.05 should be

at *P* ≤ 0.05.

**Results:**

**-**For the figures depicting mean comparisons, such as Figures 1, 3, and 4, I would recommend adding the following statement in the figure captions:

"Different lowercase letters above the bars indicate statistically significant differences between treatments at P ≤ 0.05."

- When presenting the data on ion concentrations in the plant tissues, you could include a brief comparison of the patterns observed between the two rice cultivars.

**Discussion:**

- In the first paragraph, you could start by briefly summarizing the key findings of the study before delving into the discussion.

- Consider adding a paragraph or two to discuss the potential practical implications of your findings for the management of saline water irrigation in rice cultivation.

**References:**

- The reference list appears comprehensive and up-to-date, covering the relevant literature on the topic. However, you may want to double-check the formatting of the references to ensure they adhere to the PLOS ONE style guide.

- Additionally, consider adding a few more recent references (published within the last 2-3 years) to further strengthen the context and relevance of your study within the current state of knowledge in this field.
