# Supplementary material for: Rice growth and yield responses to saline water irrigation are related to Na+/K+ ratio in plants
Source: PLoS One. 2024 Nov 1;19(11):e0312372. doi: 10.1371/journal.pone.0312372 (PMC11530064; doi:10.1371/journal.pone.0312372)
Supplement: Attachment — Submitted filename: Response to Reviewers.docx [file pone.0312372.s003.docx]

**Response to Editor and reviewer’s comments**

PONE-D-24-29091

**Rice growth and yield responses to saline water irrigation are caused by increased ion concentration in straw and roots**

DEAR EDITOR,

THANK YOU AND THE RESPECTED REVIEWERS VERY MUCH FOR THE RECOMMENDATIONS AND REVIEW COMMENTS OF OUR PAPER. PLEASE FIND BELOW OUR RESPONSES TO THE RECOMMENDATIONS AND REVIEW COMMENTS:

**Journal Requirements:**

1. When submitting your revision, we need you to address these additional requirements.

Please ensure that your manuscript meets PLOS ONE's style requirements, including those for file naming. The PLOS ONE style templates can be found at 
https://journals.plos.org/plosone/s/file?id=wjVg/PLOSOne_formatting_sample_main_body.pdf and 
<https://journals.plos.org/plosone/s/file?id=ba62/PLOSOne_formatting_sample_title_authors_affiliations.pdf>

**RESPONSE:**

THE MANUSCRIPT HAS NOW BEEN ORGANIZED MAINTAINING PLOS ONE’S STYLE REQUIREMENTS.

2. We suggest you thoroughly copyedit your manuscript for language usage, spelling, and grammar. If you do not know anyone who can help you do this, you may wish to consider employing a professional scientific editing service.

The American Journal Experts (AJE) (https://www.aje.com/) is one such service that has extensive experience helping authors meet PLOS guidelines and can provide language editing, translation, manuscript formatting, and figure formatting to ensure your manuscript meets our submission guidelines. Please note that having the manuscript copyedited by AJE or any other editing services does not guarantee selection for peer review or acceptance for publication.

Upon resubmission, please provide the following:
 
The name of the colleague or the details of the professional service that edited your manuscript

A copy of your manuscript showing your changes by either highlighting them or using track changes (uploaded as a *supporting information* file)

A clean copy of the edited manuscript (uploaded as the new *manuscript* file)

**RESPONSE:**

OUR AUTHORS PROFESSOR DR. RICHARD BELL, MURDOCH UNIVERSITY, PERTH AUSTRALIA CHECKED THOROUGHLY COPYEDIT OF OUR MANUSCRIPT FOR LANGUAGE USAGE, SPELLING, AND GRAMMAR.

3. Thank you for stating the following in the Acknowledgments Section of your manuscript: 
We would like to give thanks to Soil Science and Agronomy Division laboratory and Irrigation and Water Management Division, Bangladesh Rice Research Institute, Bangladesh for their facility to analyse soil and plant samples. We are grateful to the Ministry of Agriculture, Government of Bangladesh for funding support.

We note that you have provided funding information that is not currently declared in your Funding Statement. However, funding information should not appear in the Acknowledgments section or other areas of your manuscript. We will only publish funding information present in the Funding Statement section of the online submission form. 
Please remove any funding-related text from the manuscript and let us know how you would like to update your Funding Statement. Currently, your Funding Statement reads as follows: 
The author(s) received no specific funding for this work.

Please include your amended statements within your cover letter; we will change the online submission form on your behalf.

**RESPONSE:**

WE REMOVED THE FUNDING STATEMENT FROM THE ACKNOWLEDGEMENT SECTION. WE WOULD LIKE TO KEEP THE FUNDING STATEMENT AS SAME AS “THE AUTHOR(S) RECEIVED NO SPECIFIC FUNDING FOR THIS WORK”.

4. In the online submission form, you indicated that Data will be available on request.

All PLOS journals now require all data underlying the findings described in their manuscript to be freely available to other researchers, either a. In a public repository, b. Within the manuscript itself, or c. Uploaded as supplementary information.
This policy applies to all data except where public deposition would breach compliance with the protocol approved by your research ethics board. If your data cannot be made publicly available for ethical or legal reasons (e.g., public availability would compromise patient privacy), please explain your reasons on resubmission and your exemption request will be escalated for approval.

**RESPONSE:**

WE HAVE UPLOADED ALL DATA OF THE PRESENT STUDY AS SUPPLEMENTARY INFORMATION. THE DATA IS NOW FREELY AVAILABLE ACCORDING TO THE PLOS JOURNAL REQUIREMENTS.

5. Please include your tables as part of your main manuscript and remove the individual files. Please note that supplementary tables (should remain/ be uploaded) as separate "supporting information" files

**RESPONSE:**

WE PLACED ALL THE TABLES IN THE REVISED MANUSCRIPT.

**Reviewer 1**

1. The study effectively measures ion concentrations in both straw and roots across different salinity levels. However, the manuscript would benefit from a more detailed explanation of how the ion concentrations were measured, particularly the techniques and instruments used. This would provide greater clarity and ensure the reproducibility of the results.

**RESPONSE:**

THANK YOU VERY MUCH FOR YOUR SUGGESTIONS. ACTUALLY, WE DESCRIBED HOW TO MEASURE THE ION CONCENTRATIONS, AND THE INSTRUMENTS USED FOR THE MEASUREMENT IN THE METHODOLOGY SECTION. NOW WE ADDED MORE DETAILS OF MEASUREMENTS TECHNIQUES IN THE METHODOLOGY SECTION.

2. The research investigates the effects of saline water irrigation on two rice cultivars, yet the manuscript does not discuss the genetic or physiological basis for the observed differences between BRRI dhan67 and BRRI dhan99. A discussion on potential genetic markers or physiological traits that contribute to the salt tolerance in BRRI dhan99 would significantly enrich the findings and provide insights for future breeding programs.

**RESPONSE:**

THANK YOU FOR YOUR SUGGESTIONS. WE HAVE ADDED A PARAGRAPH IN THE DISCUSSION SECTION MENTIONING SALT TOLERANCE MECHANISM AND POTENTIALITY OF BRRI DHAN99.

3. The manuscript discusses the reduction in grain weight, straw weight, and root weight under saline water irrigation. However, the study lacks information on the statistical power of the experiments, including the sample size and the replication of treatments. It is essential to provide details on these aspects to ensure that the results are statistically robust and can be generalized beyond the specific conditions of the study.

**RESPONSE:**

THANK YOU FOR RAISING THE ISSUE. WE DID THE POT EXPERIMENT MAINTAINING ALL PROTOCOL LIKE EXPERIMENTAL DESIGN AND RANDOMIZATION. THE EXPERIMENT INCLUDES FIVE LEVELS OF SALINE WATER IRRIGATION, TWO VARIETIES AND THREE REPLICATIONS. THEREFORE, THE DEGREE OF FREEDOM WAS 8 AND THE ANOVA WAS DONE AT 95% CONFIDENCE LEVEL. WE AGREE WITH YOU THAT THE STATISTICAL POWER OF THE EXPERIMENTS DEPENDS ON SAMPLE SIZE AND SIGNIFICANCE LEVEL. MORE SAMPLE SIZE INDICATES MORE ACCURACY AND RELIABLE TEST. HOWEVER, IN THE PRESENT STUDY, THE ANOVA TEST HAD 6-10% OF CV FOR SAMPLING OF GRAIN WEIGHT AND STRAW WEIGHT. IN ADDITION, THE SIGNIFICANCE LEVEL WAS *P* < 0.1% FOR TREATMENT EFFECTS OF SALINE WATER IRRIGATION ON YIELD AND STRAW WEIGHT. THEREFORE, WE CONSIDER THAT STATISTICAL MEASURES OF THE PRESENT EXPERIMENT SHOWED VALID RESULTS.

4. The study mentions the impact of saline water on soil solute potential but does not adequately address the spatial variability of soil properties within the pots. Salinity can vary significantly within the root zone, affecting plant responses. A more rigorous approach, such as using soil moisture sensors or conducting soil sampling at different depths, would strengthen the conclusions related to soil solute potential and its impact on rice growth.

**RESPONSE:**
THANK YOU FOR YOUR OBSERVATIONS. OUR PREVIOUS STUDY IN THE SALINE AREA SHOWS THE SPATIAL VARIABILITY OF SOIL SALINITY AND MOISTURE CONTENT IN THE ROOT ZONE. IT WOULD BE BETTER IF WE COULD USE SOIL MOISTURE SENSOR AND COLLECT SOIL SAMPLES AT DIFFERENT DEPTHS. WE APOLOGIES THAT DUE TO OUR RESOURCE LIMITATIONS WE COULD NOT DO THAT. WE JUST COLLECTED SOIL SAMPLES IN ONE DEPTH (0-15 CM) AT THREE TIMES TO MEASURE SALINITY AND SOLUTE POTENTIAL. HOPEFULLY, WE WILL IMPLEMENT YOUR SUGGESTIONS IN OUR NEXT STUDY TO GET PRECISE INFORMATION.

5. The Introduction section contains several old and traditional references. To enhance the quality and relevance of the paper, I recommend updating these references with more recent and relevant citations. This will not only strengthen the background information but also align the study with the latest research developments in the field. Below are some suggested references that could be considered for inclusion:
Mitigation of salinity stress in barley genotypes with variable salt tolerance by application of zinc oxide nanoparticles
An overview of hazardous impacts of soil salinity in crops, tolerance mechanisms, and amelioration through selenium supplementation
Foliar application of ascorbic acid enhances salinity stress tolerance in barley (Hordeum vulgare L.) through modulation of morpho-physio-biochemical …
Melatonin-Induced Salinity Tolerance by Ameliorating Osmotic and Oxidative Stress in the Seedlings of Two Tomato (Solanum lycopersicum L.) Cultivars
Ameliorative Effects of Exogenous Potassium Nitrate on Antioxidant Defense System and Mineral Nutrient Uptake in Radish (Raphanus sativus L.) under Salinity …
Anatomical adaptations and ionic homeostasis in aquatic halophyte Cyperus laevigatus L. under high salinities
Structural and Functional Determinants of Physiological Pliability in Kyllinga brevifolia Rottb. for Survival in Hyper-Saline Saltmarshes
… mycoides PM35 reinforces photosynthetic efficiency, antioxidant defense, expression of stress-responsive genes, and ameliorates the effects of salinity stress in maize

**RESPONSE:**

THANK YOU VERY MUCH FOR YOUR VALUABLE SUGGESTIONS. WE HAVE INCORPORATED THE INFORMATION IN THE INTRODUCTION SECTION ACCORDING TO THE SUGGESTED REFERENCES.

**Reviewer #2:**

This manuscript is well-structured, the methods are sound, the results are comprehensive, and the discussion provides a thoughtful interpretation of the findings. The study contributes to the understanding of the physiological and ionic responses of rice to long-term saline water irrigation, which is relevant for addressing the challenges of salinity in rice production. However, I suggest minor revisions that would help to improve the clarity, flow, and overall presentation of the manuscript.

Response: Thank you very much for your encouraging positive evaluation of the study. We appreciate your kind feedback.

Title:
1. The current title "Rice growth and yield responses to saline water irrigation are caused by increased ion concentration in straw and roots" is a bit lengthy and technical. Consider a more concise and clear title, such as:

**RESPONSE:**

THANK YOU FOR YOUR SUGGESTION. WE HAVE REVISED THE MANUSCRIPT TITLE AS “RICE GROWTH AND YIELD RESPONSES TO SALINE WATER IRRIGATION ARE RELATED TO NA^+^/K^+^ RATIO IN PLANTS”.

2. Introduction:
- Consider adding a sentence or two to provide more context on the use of saline water for rice cultivation in specific regions, such as the Ganges Delta, where this study was conducted.
- You could also briefly mention the importance of understanding the physiological and ionic mechanisms behind the response of different rice cultivars to long-term saline water irrigation.

**RESPONSE:**

THANK YOU VERY MUCH FOR YOUR SUGGESTIONS. THE SALT-AFFECTED COASTAL REGION OF BANGLADESH IS THE LOWER PART OF THE GANGES DELTA WHERE MORE THAN ONE MILLION HECTARES OF LAND IS AFFECTED BY SALINITY. DRY SEASON CROP CULTIVATION IS LIMITED DUE TO SCARCITY OF FRESH WATER IN THAT REGION. THEREFORE, WE FOCUSED ON THE CONSTRAINTS OF THIS AREA AS AN EXAMPLE. WE HAVE ADDED SOME DETAILS ABOUT GANGES DELTA IN THE INTRODUCTION SECTION. ALSO, THE INTRODUCTION SECTION HAS BEEN REVISED TO INCORPORATE YOUR OTHER SUGGESTION REGARDING UNDERSTANDING THE PHYSIOLOGICAL AND IONIC MECHANISMS BEHIND THE RESPONSE OF DIFFERENT RICE CULTIVARS TO LONG-TERM SALINE WATER IRRIGATION.

3. Materials and Methods:
- in the sentence “……….were analyzed using 2-way ANOVAs” ANOVAs should be ANOVA, as it is one analysis or one design not two ANOVA designs.
- “………were separated by least significant difference (LSD) at P = 0.05 ” ) … P = 0.05 should be
at P < 0.05.

**RESPONSE:**
WE CORRECTED THE SENTENCE.

Results:
-For the figures depicting mean comparisons, such as Figures 1, 3, and 4, I would recommend adding the following statement in the figure captions:
"Different lowercase letters above the bars indicate statistically significant differences between treatments at P < 0.05."
- When presenting the data on ion concentrations in the plant tissues, you could include a brief comparison of the patterns observed between the two rice cultivars.

**RESPONSE:**

THANK YOU VERY MUCH FOR YOUR SUGGESTIONS. WE REVISED FIGURES 1,3 AND 4 CAPTIONS.

Discussion:
- In the first paragraph, you could start by briefly summarizing the key findings of the study before delving into the discussion.
- Consider adding a paragraph or two to discuss the potential practical implications of your findings for the management of saline water irrigation in rice cultivation.

**RESPONSE:**

THE DISCUSSION SECTION HAS NOW BEEN REVISED THOROUGHLY ACCORDING TO YOUR SUGGESTION.

References:
- The reference list appears comprehensive and up-to-date, covering the relevant literature on the topic. However, you may want to double-check the formatting of the references to ensure they adhere to the PLOS ONE style guide.
- Additionally, consider adding a few more recent references (published within the last 2-3 years) to further strengthen the context and relevance of your study within the current state of knowledge in this field.

**RESPONSE:**

THANK YOU FOR YOUR SUGGESTIONS. WE CHECKED THE CITATION IN THE TEXT AND IN THE REFERENCE LIST. WE ALSO ORGANIZED THE REFERENCE LIST ACCORDING TO PLOS ONE STYLE. FEW RECENT REFERENCES WERE CITED IN THE REVISED MANUSCRIPT.

**Reviewer #3:**

**Reviewer #3:**

This MS has very limited novelty and its very basic experiment. A lot of published work is available online. I have some questions about this:
1. How do different levels of saline water irrigation (EC 4, 6, 8, and 10 dS m-1) affect the growth parameters such as grain weight, dry straw weight, and root weight in two rice cultivars (BRRI dhan67 and BRRI dhan99)?

**RESPONSE:**

THANK YOU FOR YOUR INQUIRY. IN OUR PRESENT STUDY, THE TREATMENTS WERE FIVE LEVELS OF SALINE WATER AND TWO VARIETIES WITH THREE REPLICATIONS. THE ANOVA TEST SHOWED THE SIGNIFICANT DIFFERENCE OF MAIN EFFECTS (SALINE WATER AND VARIETY) ON GRAIN WEIGHT, DRY STRAW WEIGHT, AND ROOT WEIGHT. THE RESULTS ARE SHOWN IN TABLE 1 AND FIGURE 1.

2. What makes BRRI dhan99 less affected by increasing water salinity compared to BRRI dhan67? Are there specific physiological or genetic traits that contribute to its tolerance?

**RESPONSE:**

YES, BRRI DHAN99 IS MORE SALT-TOLERANT THAN BRRI DHAN67 BECAUSE OF THE ABILITY TO EXCLUSION OF ION FROM ROOT TO LEAF AND COMPARTMENTALIZATION MECHANISMS. WE HAVE ADDED DETAILED INFORMATION INTO THE DISCUSSION SECTION.

3. How does prolonged saline water irrigation influence ion concentrations (Na+, Cl-, K+) in the straw and root of rice plants, and what are the implications for soil salinity and soil solute potential?

**RESPONSE:**

THE STUDY REVEALED THAT PROLONGED SALINE WATER IRRIGATION INCREASED THE ION CONCENTRATION IN THE RICE STRAW AND ROOTS (NA^+^ AND CL^-1^). CONTINUOUS SALINE WATER IRRIGATION ALSO INCREASED SOIL SALT ACCUMULATION AND LOWERED THE SOIL SOLUTE POTENTIAL (PLEASE SEE FIG. 2). THE PRESENT STUDY SHOWED THAT IRRIGATION WATER SALINITY UP TO 4 DS M^-1^ HAD MINIMAL LOSS OF GRAIN YIELD AND RESULTED IN LESS SALT ACCUMULATION IN THE SOIL. WE DID THE SAME STUDY IN THE FIELD CONDITION (RESULT NOT YET PUBLISHED) AND FOUND THAT SALT-TOLERANT RICE VARIETIES BRRI DHAN67 AND BRRI DHAN99 HAD SIMILAR YIELD BETWEEN CONTROL (IRRIGATION WATER SALINITY 1-2 DS M^-1^) AND IRRIGATION WATER SALINITY AT 4 DS M^-1^.

4. What is the relationship between the Na+/K+ ratio in the straw and the grain weight of rice? How does this ratio serve as a potential indicator of rice yield under saline conditions?

**RESPONSE:**

OUR RESULT SHOWED THAT INCREASED WATER SALINITY HAD HIGHER NA^+^ AND LOWER K^+^ CONCENTRATIONS IN THE PLANT STRAW (PLEASE SEE FIG. 2A AND C) WHICH CONTRIBUTED TO A HIGHER NA^+^ /K^+^ RATIO (PLEASE SEE FIG. 3E). THE CORRELATION BETWEEN IONS AND GRAIN YIELD INDICATED THAT THE YIELD REDUCTION WAS HIGHLY CORRELATED WITH THE RATIO OF NA^+^/K^+^ (PLEASE SEE FIG. 5C).

5. Why does Na+ accumulate more in the straw than in the root, while Cl- accumulates more in the root than in the straw? What mechanisms are responsible for this differential ion distribution?

**RESPONSE:**

THE NA⁺ TENDS TO ACCUMULATE MORE IN THE STRAW DUE TO ITS TRANSPORT VIA THE TRANSPIRATION STREAM AND SUBSEQUENT SEQUESTRATION IN THE VACUOLES OF SHOOT CELLS. THE CL⁻, ON THE OTHER HAND, IS MORE EFFECTIVELY RETAINED IN THE ROOTS DUE TO SELECTIVE TRANSPORT AND COMPARTMENTALIZATION MECHANISMS THAT PREVENT ITS EXCESSIVE ACCUMULATION IN THE AERIAL PARTS. THESE MECHANISMS HELP THE PLANT MANAGE ION TOXICITY AND MAINTAIN IONIC BALANCE UNDER SALINE CONDITIONS.

6. How does the accumulation of salts in the soil from saline water irrigation affect the soil's physical and chemical properties, particularly soil salinity and solute potential?

**RESPONSE:**

THE ACCUMULATION OF SALTS IN THE SOIL FROM SALINE WATER IRRIGATION SIGNIFICANTLY IMPACTS BOTH THE PHYSICAL AND CHEMICAL PROPERTIES OF THE SOIL, PARTICULARLY SOIL SALINITY AND SOLUTE POTENTIAL. THE ACCUMULATION OF SALTS IN THE SOIL DUE TO SALINE WATER IRRIGATION PRIMARILY LEADS TO INCREASED SOIL SALINITY, A DECREASE IN SOLUTE POTENTIAL, AND SIGNIFICANT DEGRADATION OF SOIL STRUCTURE.

7. How accurately can the Na+/K+ ratio, Na+ concentration, and Cl- concentration be used to predict grain yield in rice under saline conditions? What are the implications for breeding programs focused on salinity tolerance?

**RESPONSE:**

THE NA⁺/K⁺ RATIO, NA⁺ CONCENTRATION, AND CL⁻ CONCENTRATION ARE IMPORTANT PHYSIOLOGICAL MARKERS THAT CAN BE USED TO PREDICT GRAIN YIELD IN RICE UNDER SALINE CONDITIONS. THE NA⁺/K⁺ RATIO IS A HIGHLY ACCURATE PREDICTOR OF GRAIN YIELD IN RICE UNDER SALINE CONDITIONS, WHILE NA⁺ AND CL⁻ CONCENTRATIONS PROVIDE VALUABLE BUT SOMEWHAT LESS PREDICTIVE INFORMATION. FOR BREEDING PROGRAMS, FOCUSING ON GENOTYPES THAT MAINTAIN A LOW NA⁺/K⁺ RATIO UNDER SALT STRESS, ALONG WITH OTHER COMPLEMENTARY TRAITS, IS KEY TO DEVELOPING SALT-TOLERANT RICE VARIETIES WITH STABLE GRAIN YIELDS. INTEGRATING THESE PHYSIOLOGICAL MARKERS INTO BREEDING STRATEGIES CAN ENHANCE THE EFFECTIVENESS OF SELECTING SALINITY TOLERANCE.

8. What are the critical salinity thresholds (dS m-1) beyond which rice growth and yield are significantly compromised, and how do these thresholds vary between the two rice cultivars studied?

**RESPONSE:**

RICE GROWTH TYPICALLY BEGINS TO BE COMPROMISED AT SALINITY LEVELS OF 3-4 DS M⁻¹, WITH SIGNIFICANT YIELD LOSSES OCCURRING BEYOND 4-5 DS M⁻¹. HOWEVER, THESE THRESHOLDS VARY BETWEEN CULTIVARS, WITH SALT-TOLERANT CAPABLE OF WITHSTANDING HIGHER SALINITY LEVELS. OUR STUDY SHOWED THAT YIELD LOSS WAS HIGHER IN BRRI DHAN67 THAN IN BRRI DHAN99 DUE TO SALINE WATER IRRIGATION (PLEASE SEE TABLE 1). HOWEVER, THIS RESULT MAY VARY IN FIELD CONDITIONS BECAUSE OF OTHER FACTORS LIKE PRESENCE OF SOIL SALINITY AND TEMPERATURE. ALSO, THE PRESENT FINDINGS INDICATE THAT LESS TOLERANCE CULTIVARS ARE AFFECTED MORE EVEN AT LOWER SALINITY LEVEL.

9. What are the long-term effects of saline water irrigation on rice yield and soil health if such practices are continued across multiple growing seasons?

**RESPONSE:**

THE PRESENT STUDY HAS BEEN IMPLEMENTED IN POT CULTURE. THE RESULTS SHOWED HOW SALINE WATER IRRIGATION AFFECTS CROP GROWTH AND YIELD, AND SOIL PROPERTIES. THE LONG-TERM USE OF SALINE WATER FOR IRRIGATION CAN HAVE DETRIMENTAL EFFECTS ON BOTH RICE YIELD AND SOIL HEALTH IF CONTINUED ACROSS MULTIPLE GROWING SEASONS. THESE EFFECTS BECOME INCREASINGLY SEVERE OVER TIME AS SALT ACCUMULATES IN THE SOIL, LEADING TO A RANGE OF AGRONOMIC AND ENVIRONMENTAL CHALLENGES.

10. How can the findings of this study be applied to improve rice cultivation practices in regions with high salinity, and what management strategies can be recommended to mitigate the negative effects of salinity on rice production.

**RESPONSE:**

THE PRESENT STUDY CONFIRMS HOW TO SUSTAIN RICE PRODUCTION AND SOIL HEALTH OVER MULTIPLE GROWING SEASONS UNDER SALINE CONDITIONS. SOME STRATEGIES MIGHT BE EFFECTIVE TO MITIGATE THE NEGATIVE EFFECTS OF SALINITY ON RICE PRODUCTION. FOR EXAMPLE, CONJUNCTIVE USE OF FRESH AND SALINE WATER, CREATING DRAINAGE FACILITY, MONITOR SOIL CONDITIONS REGULARLY, AND CONSIDER THE ADOPTION OF SALT-TOLERANT RICE VARIETIES AND SOIL REMEDIATION TECHNIQUES (GYPSUM AMENDMENT AND RICE STRAW INCORPORATION) WOULD BE USEFUL TO OVERCOME ADVERSE IMPACTS OF SALINITY AND TO DO SUCCESSFUL RICE CULTIVATION.
